# High-Efficiency CRISPR/Cas9-Mediated Gene Editing in Honeybee (*Apis mellifera*) Embryos

**DOI:** 10.1534/g3.119.400130

**Published:** 2019-04-04

**Authors:** Xiao Fen Hu, Bo Zhang, Chun Hua Liao, Zhi Jiang Zeng

**Affiliations:** Honeybee Research Institute, Jiangxi Agricultural University, Nanchang, Jiangxi 330045, China

**Keywords:** honeybee, CRISPR/Cas9, gene editing, biallelic knockout

## Abstract

The honeybee (*Apis mellifera*) is an important insect pollinator of wild flowers and crops, playing critical roles in the global ecosystem. Additionally, the honeybee serves as an ideal social insect model. Therefore, functional studies on honeybee genes are of great interest. However, until now, effective gene manipulation methods have not been available in honeybees. Here, we reported an improved CRISPR/Cas9 gene-editing method by microinjecting sgRNA and Cas9 protein into the region of zygote formation within 2 hr after queen oviposition, which allows one-step generation of biallelic knockout mutants in honeybee with high efficiency. We first targeted the *Mrjp1* gene. Two batches of honeybee embryos were collected and injected with *Mrjp1* sgRNA and Cas9 protein at the ventral cephalic side and the dorsal posterior side of the embryos, respectively. The gene-editing rate at the ventral cephalic side was 93.3%, which was much higher than that (11.8%) of the dorsal-posterior-side injection. To validate the high efficiency of our honeybee gene-editing system, we targeted another gene, *Pax6*, and injected *Pax6* sgRNA and Cas9 protein at the ventral cephalic side in the third batch. A 100% editing rate was obtained. Sanger sequencing of the TA clones showed that 73.3% (for *Mrjp1*) and 76.9% (for *Pax6*) of the edited current-generation embryos were biallelic knockout mutants. These results suggest that the CRISPR/Cas9 method we established permits one-step biallelic knockout of target genes in honeybee embryos, thereby demonstrating an efficient application to functional studies of honeybee genes. It also provides a useful reference to gene editing in other insects with elongated eggs.

The honeybee *Apis mellifera* is an important pollinator of wild flowers and crops, playing a great impact on plant diversity in the ecological environment (Aizen and Harder 2009; Klein *et al.* 2007). Honeybees have complex social behaviors including communication of food source locations via waggle dances, task specialization of colony members and group responding to environmental perturbations (Bonabeau *et al.* 1997; Franks *et al.* 2002; Williams *et al.* 2010). Therefore, the honeybee is often used as a model organism for the studies on insect social organization and behavior, physiology and development, molecular nerve mechanism, and insect genetics (Winston 1991; Menzel and Muller 1996; Caron and Connor 2013; Cridge *et al.* 2017). To understand these interesting features of honeybees, many functional genes have been studied using different molecular biological techniques, including RNA interference (Beye *et al.* 2002; Dearden *et al.* 2009), *in situ* hybridization (Fleig *et al.* 1988; Osborne and Dearden 2005; Walldorf *et al.* 1989), immunohistochemistry (Fleig 1990), transgenesis with the transposon piggyBac (Schulte *et al.* 2014), and genome editing by the clustered regular interspaced palindromic repeats (CRISPR)-associated protein (Cas9) (Kohno *et al.* 2016; Kohno and Kubo 2018).

Currently, CRISPR/Cas9-mediated gene editing has been widely used in many different species (Belhaj *et al.* 2013; Gratz *et al.* 2013; Hwang *et al.* 2013; Jiang *et al.* 2013) to understand the function of target genes due to its easy operation and high efficiency. The first application of the CRISPR/Cas9 system in arthropods was in the model insect fruit fly *Drosophila melanogaster* (Gratz *et al.* 2013) in 2013, then followed by silk worm *Bombyx mori* (Wang *et al.* 2013) in the same year; afterward, this system was used in other diverse insects including mosquito (Dong *et al.* 2015), jewel wasp (Li *et al.* 2017), butterfly (Perry *et al.* 2016) and honeybee (Kohno *et al.* 2016; Kohno and Kubo 2018). However, the CRISPR/Cas9 editing efficiency in insects was generally lower than that in mammals (Sun *et al.* 2017). There have been almost no reports of biallelic knockout mutants in insect gene-editing; usually mosaic mutants were produced, and multiple-generation breeding procedure should be conducted to produce homozygous knockout mutants, especially in the case of social insects with elongated eggs.

We speculated previous gene editing in most insects did not directly target the zygotes, as it did in mammals, instead targeting the embryonic primordial germ cells. We believed that editing efficiency would be greatly improved if the insect zygotes were exactly targeted and gene editing were performed at the correct time. Therefore, how to determine the exact region of zygote formation and suitable injection time is the key to achieve a high efficiency of gene editing in insects.

Since the latter half of the 19^th^ Century, the studies on honeybee embryonic morphology have been carried out (Cridge *et al.* 2017). A plenty of morphological data of honeybee embryos was provided. Most importantly, ten standard stages of honeybee embryos were defined (DuPraw 1967) and have been widely used to sort random and untimed embryos from any colony. Stage one (about 4.5 hr) was defined as the period begins with oviposition and ends when the cleavage nuclei initiate their migration toward the posterior pole. In the begin of stage one, newly laid honeybee egg was in the course of first meiotic division (Nachtsheim 1913; DuPraw 1967), and the center of cell division is located near the cephalic pole toward the ventral side of the egg. After about 2 hr, the ovum complete its maturation and is fertilized with sperm to form zygote. This physiological process provides the possibility of gene editing at the course of zygote formation using honeybee embryos.

Hitherto, three Cas9 deliveries of plasmid, mRNA and protein were developed and used in the arthropod organisms (Sun *et al.* 2017). Injecting the complex of sgRNA and Cas9 protein at the region of zygote formation allows Cas9 nuclease to edit genes immediately after injection. Both of Cas9 plasmid and mRNA could not function immediately (Kouranova *et al.* 2016); they need a certain time to experience a protein synthesis process in the embryo (Kouranova *et al.* 2016) and may miss the most suitable editing time. In this present study, we injected sgRNA + Cas9 protein complex into the cleavage center of honeybee embryos during the early zygote formation, we finally obtained one-step biallelic knockout honeybee embryos. This improved CRISPR/Cas9 editing system is efficient in honeybee gene-editing and can provide a solid foundation for gene functional studies in the honeybees.

## Materials and Methods

### Sample collection and Embryo slice making

The embryo samples of *Apis mellifera* were obtained from honeybee colonies maintained in the apiary of the Honeybee Research Institute, Jiangxi Agricultural University, China (28.46 °N, 115.49 °E). To collect age-controlled embryos, a mated egg-laying queen was first held in queen cages with the size of 5 cm × 3 cm × 1.5 cm for 2-3 hr, and then placed in the egg- and brood-free areas of a movable built-up comb; before the queen was placed, the built-up comb was cleaned overnight by worker bees. During queen ovulation, the queen was confined to the free space of half a comb (containing a total of 1024 cells) by a wooden or bamboo fence that only allows worker bees come in and out but does not allow the queen out. In the process of limiting the queen, the operation of the queen and the colony was as gentle as possible, so as not to frighten the queen and the worker bees. The whole process was carried out in quiet conditions. After two hours, the queen was released.

We collected all fresh *Apis mellifera* embryos (usually 30-40 embryos) within 2 hr after queen oviposition and used ten embryos to make sections. Ten remained embryos each time were taken and incubated in the incubator at 35° and 85% humidity for 4 and 6 hr respectively, and were used for making sections. All collected 10 eggs were fixed in 4% paraformaldehyde for 24 hr and then transferred into 20% sucrose solution for 48-hour dehydration at 4° ready for making sections. The dehydrated embryos were embedded in optimal cutting temperature (OCT) compound and then cut into 7-µm sections and placed on glass slides. Each embryo section was stained by propidium iodide (PI) with a concentration of 50 µg/mL and was immediately observed using a fluorescence microscope (Leica, Germany) as described in our previous report (Hu *et al.* 2018).

### Preparation of single-guide RNA and Cas9 protein

In this study, *Mrjp1* and *Pax6* genes were selected as target genes for gene editing. The sequence of *Mrjp1* sgRNA target site was 5′-TTGTTTATGCTGGTATGCCTTGG-3′ (Underlined is the PAM sequence), which was designed according to the previous report and located on the exon 2 of *Mrjp1* gene (Kohno *et al.* 2016). For *Pax6* gene, we identified an sgRNA target site (5′-GACCATTACCAGACTCTACAAGG-3′; form +1106 to +1128 on the reference sequence of XM_006565377.2) in exon 2 of *Pax6* using the CCTop online tool (Stemmer *et al.* 2015; Stemmer *et al.* 2017). A PCR-based approach was used to produce sgRNAs of *Mrjp1* and *Pax6*. A specific oligonucleotide encoding a T7 polymerase-binding site and the sgRNA target sequences of *Mrjp1* or *Pax6* was designed as the forward primers (F-sgRNAMrjp1: 5′-TAATACGACTCACTATAGTTGTTTATGCTGGTATGCCTgttttagagctagaaatagc-3′ for *Mrjp1*; F-sgRNAPax6: 5′-TAATACGACTCACTATAGACCATTACCAGACTCTACAgttttagagctagaaatagc-3′ for *Pax6*) and a common oligonucleotide encoding the remaining sgRNA sequences was designed as the reverse primer (R-Common: 5′-AAAAAAAGCACCGACTCGGTGCCAC-3′). The two pairs of primers for *Mrjp1* and *Pax6* were annealed by PCR to synthesize template DNA. The PCR reaction mixture (40 μl) contained 20 μl of 2 × Pfu Mastermix (Transgene), 14 μl of H_2_O, 2 μl of 5 μM forward and reverse primers (F-sgRNAMrjp1 or F-sgRNAPax6 and R-Common) and 2 μl of 20 ng/μl pYSY-sgRNA plasmid (YaoShunYu, China). PCR was performed at 95° 3 min, 30 cycles of (95° 30 s, 56° 30 s, 72° 30 s), 72° 10 min and 12° ∞. PCR products were purified by a PCR clean-up (Axygen) kit. *In vitro* transcription was performed with the HighMAXIscript SP6/T7 RNA *in vitro* transcription kit (Ambion, USA) according to the manufacturer’s instruction.

The Cas9 protein (TrueCut Cas9 Protein v2) was purchased from Thermo Fisher Scientific (Shanghai, China).

### Microinjection and rearing

A honeybee queen was placed in a quiet, clean and egg-free comb, and allowed to lay eggs. After two hours, the queen was released. The plastic plugs together with the laid eggs were taken off from the built-up comb, and the attached eggs were ready for injection.

We used a microinjection device (Eppendorf FemtoJet) and an Oxford micromanipulator (Eppendorf TransferMan NK2) to inject the embryos under an inverted microscope. One delivery of 200 ng/μl *Mrjp1* sgRNA and 200 ng/μl Cas9 protein was injected into honeybee embryos from the m_1_ (n = 24) and m_2_ (n = 26) batches at the sites of the ventral side near to embryonic cephalic pole (the region of zygote formation) and the dorsal posterior side of the embryos, respectively. Another delivery of 200 ng/μl *Pax6* sgRNA and 200 ng/μl Cas9 protein was injected into honeybee embryos from the p_3_ (n = 22) batch at the sites of the ventral side near to the embryonic cephalic pole. Before injection, the solution of sgRNA and Cas9 protein was mixed well and then placed on ice for half an hour. The tips of the injection needles were rigid and the internal diameter of the glass needle tip was 4 μm. When we performed the injection operation, the angle between the needle and the side of the egg was adjusted to less than 30 degrees and the needle was inserted following the direction from the anterior side to the posterior side. Once the tip of the needle entered the egg, injection took place, avoiding deep insertion. The injection time was 0.1 s, the injection pressure was 600 hPa, and the balance pressure was 50 hPa. The injection parameters were similar to the previous description (Schulte *et al.* 2014). The integrity of the egg should be ensured after injection. Eggs with protoplasm overflowing were removed, as they would die because of damage. A transparent spot was found at the injection site of the egg, which then disappeared slowly.

The embryos were incubated in plastic boxes at 35° (relative humidity 85%) with a small amount of 16% (vol/vol) sulfuric acid to prevent mold formation (Schulte *et al.* 2014).

### Pre-sequencing process of mutants

After microinjection, the eggs were incubated for 48-60 hr. Then the embryonic morphology was observed under the microscope. At this time, the normally developed egg is in the ninth stage of embryonic development, and there is a very obvious fluid-filled gap in the anterior pole and a protuberance at the head of the amnion-serosa (Figure S1), which can be used as a criterion for selection. Embryos with normal developmental morphogenesis were used as the materials for PCR analysis of knockout target genes. PCRs for target genes were performed following the instructions of the TransDirect Animal Tissue PCR Kit purchased from Transgen Biotech (Beijing, China).

The fragment (403 bp) flanking the editing target site in *Mrjp1* was amplified using a pair of specific primers (forward: 5′-ATATTCCATTGCTTCGTTACTCG-3′, reverse: 5′-TGGATATGAAGAATTTTGGACAAG-3′). The fragment (446bp) flanking the editing target site in *Pax6* was amplified using another pair of specific primers (forward: 5′-GCCGGTGTGTGTTTATTCAA-3′, reverse: 5′- TGCAAAAGTGACATCCTTGC T-3′). The PCR reaction mixture (20 μl) contained 10 μl of 2 × TransDirect PCR supermix (+dye), 0.4 μl of forward primers, 0.4 μl of reverse primers, 5.2 μl of ddH_2_O, 4 μl of embryonic lysis fluid. PCR was performed at 94° 10min, 35 cycles of (94° 30 s, 52° 30 s, 72° 1min), 72° 10 min and 12° until the PCR products were taken out. The PCR products were ready for Sanger sequencing or inserted into TA vectors for Sanger sequencing.

### PCR of negative controls

Honeybees used in this experiment came from a common *Apis mellifera* colony without special breeding. To better determine the type of knockout mutants, we investigated the flanking sequences around the PAM sites of *Mrjp1* and *Pax6* genes in the unedited embryos laid by the experimental queens. For each gene, ten unedited embryos were collected and PCR amplification was performed same as the corresponding candidate genes. The PCR products were used as negative controls for Sanger sequencing or TA cloning.

### Sequencing of PCR products and TA clones

Sanger sequencing was conducted using the forward primers of *Mrjp1* or *Pax6* as the sequencing primer. Different single sequencing peak from wild-type samples and double sequencing peaks present as a cluster of bases indicated a mutation event. All PCR products, except the samples with obvious clean single peaks, were TA-cloned, and 20 colonies were collected for Sanger sequencing by Tsingke Biological Technology (Changsha, China) to determine the exact indel types.

### Data and reagent availability

The authors state that all data necessary for confirming the conclusions presented in the article are represented fully within the figures and the tables. All honeybee strains and reagents are available upon request. Supplemental material available at Figshare: Figure S1: https://figshare.com/s/5fb9705f6599cb0648fb, Figure S2: https://figshare.com/s/341d52e9e01fc5948fb3, Figure S3: https://figshare.com/s/b42356ad88a71aba15d0, Figure S4: https://figshare.com/s/b5219e7639974f11aca6.

## Results

### Morphological slice analysis of early honeybee embryos

**Figure 1:**
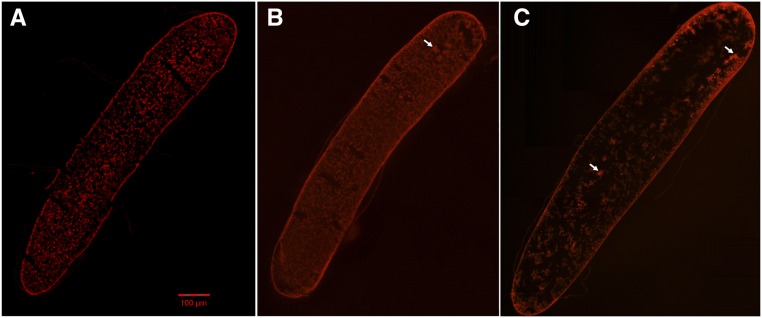
Embryo slices for the 0-2, 4-6 and 6-8 h samples. The white arrow denotes the energid. Scale bar = 100 μm.

To determine reasonable injection site and time for the gene editing, we first analyzed morphological slices of early honeybee embryos at the ages of approximate 0-2, 4-6 and 6-8 hr (h). From the slice of 0-2 h honeybee embryo (Figure 1A), we observed that the homogeneous protoplasm occupied the whole embryo and there was relatively little structure. We could not find any obvious cleavage nuclei with deeper staining. According to the description of DuPraw (1967), the egg of this period is still in the course of zygote formation which is near the cephalic pole toward the ventral side of the egg, so the energid with deep staining could not be observed on a slice. As shown in the slice of 4-6 h honeybee embryo (Figure 1B), several energids, formed by nuclei recruiting their own plasm islands, were found together with deeper staining near the anterior pole of embryo. It was a typical structure of late stage one or early stage two embryo, which was consistent with the morphology descripted by DuPraw (1967): “At the beginning of stage 2 a cluster of eight energids lies near the 10% level (measured from the anterior pole), about equidistant from the dorsal, ventral, and laternal surfaces”. The 6-8 hr honeybee embryos (Figure 1C) were in the middle or late stage two of embryonic development. Many energids were found but not clustered together in the cleavage center. All energids were migrating dispersedly toward the surface of embryo and some had reached the surface of anterior pole.

These embryonic slices convinced us that zygote formation happens in the region near the cephalic pole toward the ventral side of the egg around 2 hr after queen oviposition.

### Mutagenesis of Mrjp1 gene by CRISPR/Cas9

**Figure 2:**
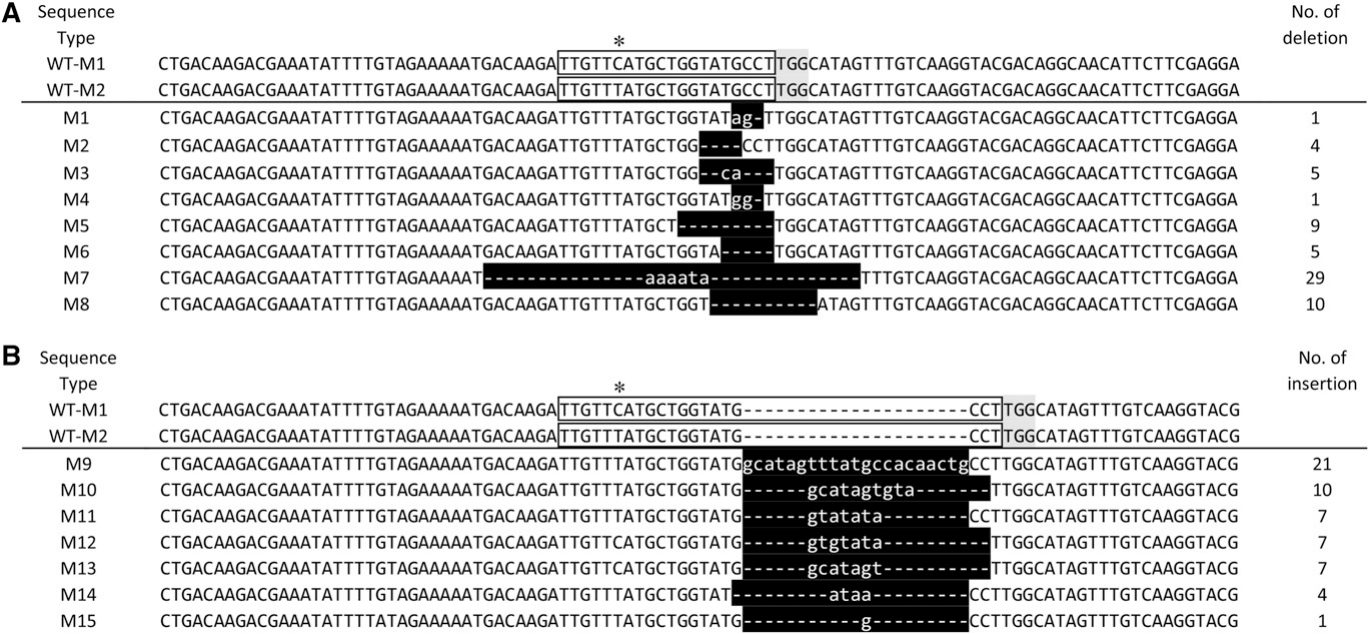
Gene editing patterns of Mrjp1 gene. A deletion; B insertion. The two sequences shown at the top are wild-type sequences of WT-M1 and WT-M2. Letters in gray boxes and in blank boxes with a black frame indicate PAM and sgRNA target site, respectively. Star indicates SNP of *T/C* in wild-type sequence. White letters and dashes in the black boxes indicate inserted and deleted nucleotide sequences, respectively. Sequence type from which each sequence type was detected are shown on the left. The numbers of nucleotide deletions or insertion that differed between the genome-edited embryos and wild-type (WT) sequences are shown on the right.

In the m_1_ and m_2_ batches, 17 and 15 normal embryos were obtained; the normal embryonic development rates were 65.4% and 62.5%, respectively (Table 1). The PCR products of all these embryos were obtained. The editing types of these two batches of target genes are shown in Figure S2 and S3. We sequenced the flanking region around the PAM site of *Mrjp1* gene in 10 blank samples and found a *T/C* mutation at the sgRNA site of *Mrjp1* gene (5′-TTGTTTATGCTGTA(T/C)GCCTTGG-3′, Figure 2A), which was not in the core recognition region of *Mrjp1* sgRNA.

**Table 1:** Differences in gene editing efficiency of *Mrjp1* or *Pax6* at the different injection sites

Batch	Target gene	Injection site	Total	Survival^1^ (%)	Mutated (%)	Biallelic knockout	
						Non-mosaic (%)	Mosaic (%)
**m_1_**	*Mrjp1*	Dorsal posterior side	26	17 (65.4%)	2 (11.8%)	0	0
**m_2_**	*Mrjp1*	Ventral cephalic side	24	15 (62.5%)	14 (93.3%)	5 (33.3%)	6 (40.0%)
**p_3_**	*Pax6*	Ventral cephalic side	22	13 (59.1%)	13 (100.0%)	3 (23.1%)	7 (53.8%)

1 , the number of injected embryos that have developed into stage 9.

In the m_1_ batch, only two chimeric embryos, 8C and 6C, were detected among 17 injected samples (Figure S2). The results in the m_2_ batch showed that there were three types of results in the samples at the injection site of the ventral side near to embryonic cephalic pole: (1) the first type is chimera with obvious double peaks shown as the 1A sample in Figure S3, which included 8 other samples of 2A, 3A, 6A, 9A, 10A, 12A, 14A and 15A; (2) the second type is complete knockout mutant with clean single peaks different from wild-type shown as the 11A sample in Figure S3. Four other samples of 4A, 5A, 8A and 13A were included; and (3) the third type is an entirely unedited sample like 7A, only one sample belongs to this type (Figure S3).

The PCR products of 11 chimera samples (9 samples injected at the ventral-cephalic-side and 2 samples injected at the dorsal-posterior-side) from the m_1_ and m_2_ batches were inserted into TA-clones, and 20 clones were randomly selected and sequenced. The sequencing results for different types of gene editing were shown in Figure 2. We found that the editing efficiency of samples injected in the ventral side near cephalic pole (93.3%) was significantly higher than that of samples injected at the dorsal posterior side (11.8%). Overall, 73.3% of the individuals injected near the ventral side of the cephalic pole were biallelic knockout mutants (5 biallelic homozygous mutants and 6 biallelic heterozygous mutants), while only two low-editing-rate chimeras (Table 2) were obtained when injecting at the dorsal posterior side of embryos. The results showed that the editing efficiency of an injection site near the ventral side of the cephalic pole was significantly higher than that of injection site at the dorsal posterior side.

**Table 2:** The editing types of all edited individuals in three batches of experiments

Injection site	ID	Target gene	Editing type (number)	Editing Rate
**Ventral cephalic side**	1A	*Mrjp1*	Wild-type(12) M15(8)	8/20 = 40%
	2A	*Mrjp1*	Wild-type (14) M12(1) M15(5)	6/20 = 30%
	3A	*Mrjp1*	M1(14) M2(1) M3(1) M6(3) M15(1)	20/20 = 100%
	4A	*Mrjp1*	M8	100%
	5A	*Mrjp1*	M6	100%
	6A	*Mrjp1*	Wild-type(8)M8(12)	12/20 = 60%
	8A	*Mrjp1*	M6	100%
	9A	*Mrjp1*	M5(12) M8(8)	20/20 = 100%
	10A	*Mrjp1*	M4(14) M13(6)	20/20 = 100%
	11A	*Mrjp1*	M8	100%
	12A	*Mrjp1*	M11(2) M15(18)	20/20 = 100%
	13A	*Mrjp1*	M6	100%
	14A	*Mrjp1*	M8(14) M9(5) M14(1)	20/20 = 100%
	15A	*Mrjp1*	M8(10) M10(10)	20/20 = 100%
	1D	*Pax6*	Wild-type(1) P6(19)	19/20 = 95%
	2D	*Pax6*	P11	100%
	3D	*Pax6*	Wild-type(1) P7(16) P14(3)	19/20 = 95%
	4D	*Pax6*	P9(2) P16(18)	20/20 = 100%
	5D	*Pax6*	P7(16) P15(4)	20/20 = 100%
	6D	*Pax6*	P1(3) P7(17)	20/20 = 100%
	7D	*Pax6*	P4(17) P5(1) P7(1) P10(1)	20/20 = 100%
	8D	*Pax6*	P7(1) P3(19)	20/20 = 100%
	9D	*Pax6*	P7	100%
	10D	*Pax6*	P7(1) P8(1) P13(18)	20/20 = 100%
	11D	*Pax6*	P2(10) P7(1) P13(9)	20/20 = 100%
	12D	*Pax6*	P12(20)	20/20 = 100%
	13D	*Pax6*	Wild-type(1) P7(19)	19/20 = 95%
**Dorsal posterior side**	6C	*Mrjp1*	Wild-type(19) M7(1)	1/20 = 5%
	8C	*Mrjp1*	Wild-type(14) M6(6)	6/20 = 30%

Note: 4A, 5A, 8A, 11A, 13A, 2D, 9D and 12D were biallelic homozygous mutants; 3A, 9A, 10A, 12A, 14A, 15A, 4D, 5D, 6D, 7D, 8D, 10D and 11D were biallelic heterozygous mutants.

### Mutagenesis of Pax6 gene by CRISPR/Cas9

**Figure 3:**
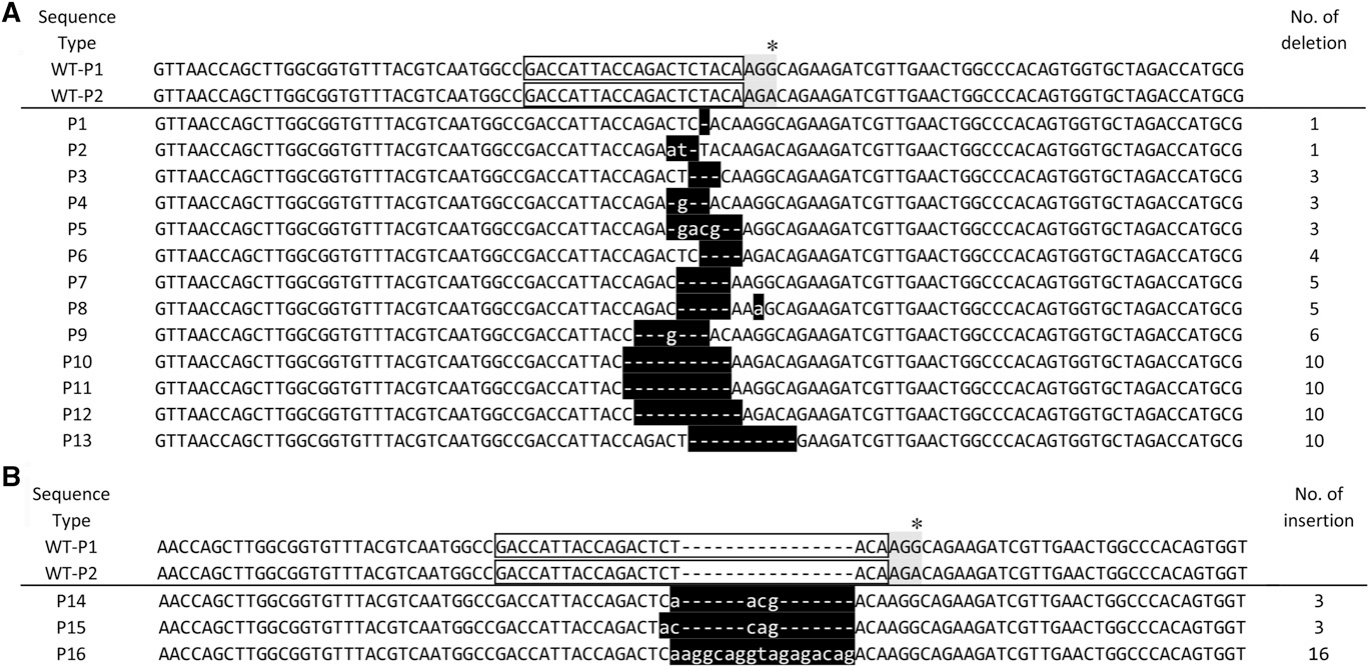
Gene editing patterns of Pax6 gene. A deletion; B insertion. The two sequences shown at the top are wild-type sequences of WT-P1 and WT-P2. Letters in gray boxes and in blank boxes with a black frame indicate PAM and sgRNA target site, respectively. Star indicates SNP of *G/A* in wild-type sequence. White letters and dashes in the black boxes indicate inserted and deleted nucleotide sequences, respectively. Sequence type from which each sequence type was detected are shown on the left. The numbers of nucleotide deletions or insertion that differed between the genome-edited embryos and wild-type (WT) sequences are shown on the right.

In the p_3_ batch, 13 normal embryos were obtained; the normal embryonic development rate was 59.1% (Table 1). The PCR products of all these embryos were obtained. The editing types of *Pax6* gene are shown in Figure S4. For *Pax6* gene, we also sequenced the target gene sequences of 10 wild-type samples, and found a *G/A* variant 5′-GACCATTACCAGACTACAAG(G/A)-3′ on the sgRNA site, which happened to be in the PAM site (see the WT-P1 and WT-P2 sequences in Figure 3), but AGA is also a non-canonical PAM site. The cutting efficiency was reported lower than AGG (Kleinstiver *et al.* 2015; Zhang *et al.* 2014), which may affect the editing efficiency.

There were three editing results for the p_3_ samples injected into the ventral site near the cephalic pole: (1) the first type is chimeras with obvious double peaks, including eight samples of 1D, 3D, 4D, 5D, 6D, 7D, 8D and 11D; (2) the second type is complete-like knockout with little weak peaks, including three samples of 10D, 12D and 13D; and (3) the third type is complete knockout with clean single peaks shown in Figure S4, including two samples of 2D and 9D. The PCR products of 11 samples from the first and second types were inserted into TA-clones, and then 20 clones for each PCR product were selected for sequencing. The sequencing results for different types of gene editing were shown in Figure 3. The results showed that the efficiency of target gene *Pax6* editing was very high, reaching 100% editing rate (Table 2). 76.9% of the individuals injected near the ventral side of the cephalic pole were biallelic knockout mutants (3 biallelic homozygous mutants and 7 biallelic heterozygous mutants).

## Discussion

CRISPR/Cas9-mediated gene-editing has achieved tremendous success and impact in many species. However, the editing efficiency in insects was not as high as that in mammals, especially for those with elongated eggs, such as honeybee (Kohno *et al.* 2016; Kohno and Kubo 2018; the rates of edited offspring were below than 12.5%) and mosquito (Dong *et al.* 2015; the knockout efficiency was 5.5%). It led to the fact that CRISPR/Cas9 technology has not been widely used in honeybees. In this study, we reported an improved CRISPR/Cas9 gene-editing method by microinjecting sgRNA and Cas9 protein into the region of zygote formation within 2 hr after queen oviposition, which allows one-step generation of biallelic knockout mutants in honeybee with high efficiency.

We first targeted *Mrjp1* gene. The design of the *Mrjp1* sgRNA was same as previous report by Kohno *et al.* (2016). The main aims of editing honeybee *Mrjp1* gene were: (1) determining injection time, (2) determining injection site, and (3) determining the feasibility of the sgRNA and Cas9 protein delivery. We wanted to know whether the ventral cephalic side (near the position of zygote formation) was more suitable for the injection site than the dorsal posterior site as previously described (Kohno *et al.* 2016), and whether the eggs at the developmental stage of zygote formation collected by our method were suitable for gene editing. Through our exploration, the results showed that the improvement in the above three aspects could help us get a high gene-editing rate in honeybees. To further demonstrate the efficiency of the improved Crispr/Cas9 editing system, we designed an sgRNA for another target gene *Pax6*. The results showed that the gene editing efficiency for *Pax6* gene was also high.

### Effect of mutation in sgRNA of target genes

We examined the chimeras (1A, 2A, 6A, 1D, 3D and 10D) in the two batches of *Mrjp1* and *Pax6* gene editing. We found that the TA clones derived from the *Mrjp1* chimera embryos share a common feature: the fifteenth base at the left flank to PAM site is the C sequence (Figure 2). The *Mrjp1* sgRNA was same to previous report originally designed based on the reference sequence of *Mrjp1* gene. However, our sequencing result of wild type embryos showed that there was a mutation of *T > C* at the fifteenth base at the left flank to PAM site. Although this mutation is not located in the core sequence of the 6-12 base near PAM (Jiang *et al.* 2013), the results show that the heterozygosity of this site has a negative impact on editing efficiency.

We designed the *Pax6* sgRNA according to the NCBI reference sequence of *Pax6* gene. However, based on the sequencing results of wild-type embryos, we found that there was a mutation of *G > A* at the PAM site (Figure S3) on the guide RNA recognition sequence. Previous study showed that both AGG and AGA are recognizable PAM sites, but the editing efficiency of AGG was higher than that of AGA (Kleinstiver *et al.* 2015; Zhang *et al.* 2014). In our case, based on the sequencing results of TA clones, all the unedited sequences (n = 3) contained the AGA PAM, which was found in only a low proportion of all tested embryos; most of sequences (n = 40) containing AGA were edited in the mutagenesis of *Pax6*. The lowest editing rate of target gene *Pax6* was 95%. So it suggested that the *Pax6* sgRNA used in our study was relatively specific.

### Improvements in our honeybee gene editing system

In this study, we have adjusted injection time, injection site, and injection delivery to honeybee embryos, with the aim of directly targeting the honeybee zygote and determining the optimum time point to perform gene editing. We have made significant improvements in the methodology of gene editing in honeybees from the technical perspective. Fortunately, we finally achieved high efficiency of gene editing in honeybee and one-step production of honeybee biallelic mutants. Therefore, we have provided a method for efficient gene-editing in honeybee, which was an obvious technological improvement compared to the previously established methodology (Kohno *et al.* 2016).

Here, we summarized three major improvements in our honeybee gene editing system as follows:

First, for honeybee, an insect with elongated embryos, gene editing at right embryonic position and at accurate embryonic developmental time is an important technological improvement and is the key to gain high efficient gene-editing results. In previous studies on honeybee genetic manipulation (Schulte *et al.* 2014; Kohno *et al.* 2016; Kohno and Kubo 2018), the position of primitive gonad cells (dorsal posterior region of embryos) was chosen for injection, which resulted in generation of mosaic queens or low efficient rate of transgenesis (27% and 20%) or gene-editing (< 12.5%). And it needed a complex breeding process to obtain the offspring mutants. However in mammals, such as mice (Wang *et al.* 2013), rats (Li *et al.* 2013) and pigs (Hai *et al.* 2014), zygote was directly targeted as the injection object of gene editing, and the biallelic knockdown mutants could be obtained at the current generation (G0 generation) by one-step injection. Therefore, we considered that targeting the honeybee zygote instead of the primitive gonad cells should be the key to achieving highly efficient gene-editing results in honeybee. Before our embryonic injection, we paid more attention to the development of honeybee embryos. The elongated egg of honeybee is greatly different from the round or nearly round egg of fruit fly, silkworm or other insects in the morphological and anatomical structure. We carefully read and understood previous literature about the development of honeybee embryo and observed histological structure of early honeybee embryos. We were sure that the course of zygote formation in honeybee embryos occurred about two hours after queen ovulation and the position of honeybee zygote formation was the site of the ventral side near to embryonic cephalic pole. In addition, to allow Cas9 nuclease to edit genes immediately after injection, we chose the complex of sgRNA and Cas9 protein as injection delivery and injected the complex into the region of honeybee zygote formation. While both Cas9 plasmid and mRNA need a certain time for protein synthesis process in the embryo (Kouranova *et al.* 2016) and may miss the suitable editing time at the 1- or 2- cell stage of honeybee zygote. Finally, we successfully achieved highly efficient gene editing in honeybee embryos. We could make a conclusion that the improvement of injection site, time and delivery resulted in high efficiency of honeybee gene editing. This method we established also provided a reference for gene editing in other insects with elongated embryos.

Second, compared to previous honeybee gene-editing methodology, the generation efficiency of mutants has been greatly improved. To our knowledge, two papers have reported CRISPR/Cas9 gene editing in honeybees from Kubo’s group (Kohno *et al.* 2016; Kohno and Kubo 2018). Their generation efficiency of honeybee mutants were 12.4%, 5.1% and 10%, respectively. While in our study, the efficiency of bi-allelic mutants for two candidate genes were 73.3% and 76.9%, respectively, which were much higher than those in the studies of Kubo’s group.

Third, one-step high-efficiency gene-editing method established in our present study could greatly accelerate the production of biallelic knockout honeybee mutants. In previous application of CRISPR/Cas9 in honeybees, the biallelic knockout honeybee mutants could only be achieved through complex breeding procedures over several generations. Usually, three generations of queens with mutated gene needed to be cultivated (Kohno *et al.* 2016; Kohno and Kubo 2018). Honeybee is a typical social insect. Honeybees have three castes: drones, workers, and queens. Drones are male, while workers and queens are female. Haploid embryos develop into drones; diploid embryos develop into worker bees or queens. Honeybee development consists of four stages: embryo, larva, pupa and adult. In the stage of larvae, there is a great difference in bee diet between the larvae developing into queens and that developing to worker bees, which is controlled by worker bees with the duty of feeding brood. In other words, the production of queen needs the feeding of nurse bees. To our knowledge, there were no reports that queens could be artificially bred without nurse bees’ feeding. It was difficult to achieve artificial breeding of queen with reproductive ability. The larvae ready to develop into queen needed to be transferred to the natural colony and fed by nurse bees. In addition, nurse bees would strictly supervise the hatching eggs and larvae in the natural colony. If there were weak embryos or damaged embryos, they would be cleaned out. The injected embryos were unavoidably damaged to a certain extent compared to the normal embryos. So there was a large probability that the injected embryos would be cleaned out by nurse bees. For these above reasons, in the process of obtaining mutant worker bees by microinjection CRISPR/Cas9 gene editing method, the developmental process from injected embryos to queens became a bottleneck problem, which easily led to the failure of the whole experiment or the great reduction of the experimental efficiency.

In our present study, we established one-step high-efficiency gene-editing method. We could obtain biallelic mutants through one generation cultivation of worker bees. Our method avoids the complex process of queen breeding and greatly reduces the difficulty of the whole experiment of honeybee gene editing. Its application in honeybees could also rapidly reveal the phenotypes of gene knockout mutants. In addition, this efficient gene editing approach provides the possibility of functional studies of genes critical for development from embryo into adult. All together, we considered that the improvement of honeybee gene-editing method was obvious from the technological perspective, and its effect and significance were remarkable.

Finally, it should be noted that we have not reared any of the edited embryos into larvae or adults. The efficacy of our method awaits testing in a full egg-to-adult system to ensure that there are no unexpected problems associated with the injection innovations during the rearing process.

### Conclusions

In the present study, we targeted the region of zygote formation at the 1-2 cell stage, injected the complex formed by sgRNA and Cas9 protein as the delivery into honeybee embryos, and carried out gene editing experiments on *Mrjp1* and *Pax6* genes. The results showed that the CRISPR/Cas9 editing system we established could produce G0 knockout mutant embryos with high efficiency in honeybees. This efficient CRISPR/Cas9 editing system paves the road for the gene functional studies in honeybees and provides a useful reference to the gene editing in other insects.
